# Towards a World Wide Web without digital inequality

**DOI:** 10.1073/pnas.2212649120

**Published:** 2023-01-09

**Authors:** Moumena Chaqfeh, Rohail Asim, Bedoor AlShebli, Muhammad Fareed Zaffar, Talal Rahwan, Yasir Zaki

**Affiliations:** ^a^Computer Science, Science Division, New York University Abu Dhabi 129188 Abu Dhabi, UAE; ^b^Social Science Division, New York University Abu Dhabi, 129188 Abu Dhabi, UAE; ^c^Syed Babar Ali School of Science and Engineering, Lahore University of Management Sciences, Lahore, Punjab 54792, Pakistan

**Keywords:** World Wide Web, digital divide, low-end mobile phone

## Abstract

The World Wide Web (WWW) empowers people in developing regions by eradicating illiteracy, supporting women, and generating economic opportunities. However, their reliance on limited bandwidth and low-end phones leaves them with a poorer browsing experience compared to privileged users across the digital divide. To evaluate the extent of this phenomenon, we sent participants to 56 cities to measure the cost of mobile data and the average page load time. We found the cost to be orders of magnitude greater, and the average page load time to be four times slower, in some locations compared to others. Analyzing how popular webpages have changed over the past years suggests that they are increasingly designed with high processing power in mind, effectively leaving the less fortunate users behind. Addressing this digital inequality through new infrastructure takes years to complete and billions of dollars to finance. A more practical solution is to make the webpages more accessible by reducing their size and optimizing their load time. To this end, we developed a solution called Lite-Web and evaluated it in the Gilgit-Baltistan province of Pakistan, demonstrating that it transforms the browsing experience of a Pakistani villager using a low-end phone to almost that of a Dubai resident using a flagship phone. A user study in two high schools in Pakistan confirms that the performance gains come at no expense to the pages’ look and functionality. These findings suggest that deploying Lite-Web at scale would constitute a major step toward a WWW without digital inequality.

## Significance

Developing regions suffer from poor Internet connection and overreliance on low-end phones, which violates net neutrality—the idea that all Internet traffic should be treated equally. We sent participants to 56 countries to measure global variation in web-browsing experience, revealing significant inequality in mobile data cost and page load time. We also show that popular webpages are increasingly tailored to high-end phones, thereby exacerbating the inequality. Our solution, Lite-Web, makes webpages faster to load and easier to process on low-end phones. Evaluating Lite-Web on the ground reveals that it transforms the browsing experience of Pakistani villagers with low-end phones to that of Dubai residents with high-end phones. These findings call attention from researchers and policy makers to mitigate digital inequality.

The World Wide Web (WWW) was envisioned as an egalitarian platform that provides universal access to the wealth of accumulated human knowledge. It has enabled the creation of projects such as Wikipedia, Khan Academy, and Massive Open Online Courses (MOOCs), all of which hold the promise of democratizing education (1). In developing regions, the WWW has contributed to women’s empowerment by offering a gender-opaque medium that alleviates bias, provides access to distance learning and employment opportunities, and increases the chances of receiving support from organizations concerned with the well-being of women (2, 3). Another way in which the WWW supports the developing regions is by generating economic opportunities. For example, it has been shown that fast Internet access can decrease (un)employment inequality in Africa (4), and mobile broadband access can decrease poverty, particularly among rural households (5). Additionally, the development of e-commerce can play a significant role in narrowing the urban–rural income gap in China (6). Access to critical information empowers farmers and fishermen in emerging markets. For example, by tracking weather conditions and comparing wholesale prices, farmers and fishermen in India increased their profit by 8%, eventually leading to a 4% decrease in prices for their customers (7). The WWW is even helping eradicate illiteracy—one of the main barriers to digital inclusion. For example, in Sub-Saharan Africa, where most people do not own any books, the massive proliferation of mobile devices allows people to develop, sustain, and enhance their literacy skills by providing a medium through which they can access reading materials (8). Moreover, providing access to online material in Malawian boarding schools can encourage reading and improve educational outcomes (9). Other examples include nonprofit initiatives such as remoteStudentExchange.org, which in the few months since its launch in January 2021 has given thousands of students in low- and middle-income countries direct access to (online) courses from the world’s leading universities.

The growing adoption of mobile phones has contributed to the significant increase in Internet access over the past decade. In 2018, nearly 300 million users were newly connected to the mobile web, and in 2019, the total number of mobile web users exceeded 3.5 billion worldwide. Of those users, 74% live in low- and middle-income countries (10), where mobile phones are the primary means of Internet access. Many of those users depend solely on mobile phones. For instance, across 18 developing countries, an average of 57% of Internet access in 2018 was carried out exclusively via mobile phones (10).

A key enabler of mobile Internet adoption is affordability; not only is mobile data becoming available at lower prices (11) but also mobile phones are becoming more affordable. For instance, cheaper phones are expected to be available in Pakistan (12), India (13), and Africa (14) in the near future, as a new generation of phones is expected to be made available for only $20 (15). Although access to the mobile web is expanding in developing countries (16) to the point of surpassing access to piped water and consistent electricity (17), the user experience remains poor (18). This is part of a larger phenomenon known as the *digital divide*, which separates those with high-quality access to information and communications technologies from those with poorer alternatives (16). Our primary goal is to evaluate the extent of this phenomenon worldwide and to explore affordable and scalable solutions that can potentially bridge the divide and alleviate the digital inequality experienced by underserved communities.

## Results

### Understanding Digital Inequality.

To better understand the variation in web access quality across the globe, we needed to send participants to different cities spanning six continents and have each of them access the same set of webpages (to control the browsing experience) using the exact same hardware (to control the processing power) and the same web browser (Google Chrome in our case) at the same local time (12:00 pm in our case) while being connected to a cellular network (rather than Wi-Fi) to ensure that any observed differences in average page load time are not influenced by variations in these factors. To this end, we leveraged the diversity of the student population at New York University Abu Dhabi by recruiting undergraduates traveling back to their home countries during the winter break. Each participant was handed the same low-end phone model, namely Xiaomi Redmi Go, and was asked to install a tool on their laptop, called WebPageTest (19), which automates web requests on that phone while recording various page load time metrics using the actual (rather than emulated) connection speed. Those web requests were for the 100 webpages that were most frequently visited worldwide at the time according to Alexa (20).

The students whom we recruited ended up visiting 72 cities across six continents. Upon their arrival at their respective destinations, participants purchased a SIM card along with an affordable pricing plan from a local service provider and kept the receipt which specified the total cost and the number of Gigabytes provided by the plan. After that, they connected the phone to their laptop via a USB cable and ran the tool at 12:00 pm local time to automatically request the 100 webpages via Google Chrome on the phone and extract the results. The experiment took place in December 2019 and January 2020. Students who failed to follow the experimental protocol were discarded from our analysis, yielding a total of 56 cities.

**Fig. 1.**
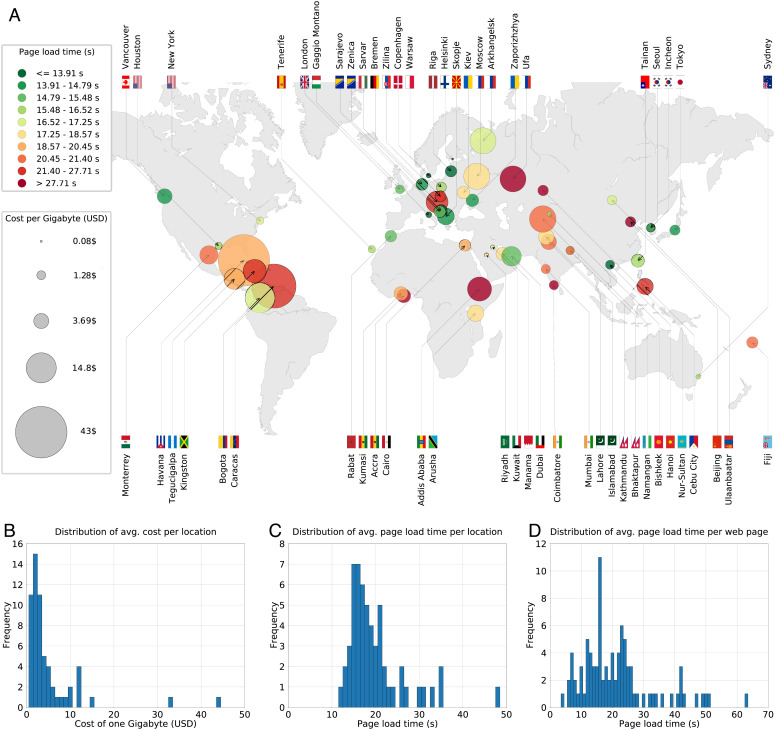
Average page load time and data cost across different locations. (*A*) Each location is represented by a circle whose diameter reflects the costs per Gigabyte (measured based on the purchasing power parity) and whose color represents the average page load time (measured in seconds) of the 100 most frequently visited pages worldwide. Page load times were measured by accessing the pages via the same low-end mobile phone model—Xiaomi Redmi Go—using a cellular network at that location. (*B*) Distribution of the cost of 1 GB (USD) per location. (*C*) Distribution of the page load time (s) per location, averaged over different webpages. (*D*) Distribution of the page load time (s) per webpage, averaged over different locations.

When comparing the price of 1 GB across countries, one needs to take into consideration the differences in living standards. For instance, even if the cost of 1 GB cost in a rich country was the same as that in a poor country (e.g., 1 USD), this may be considered affordable in the former but not the latter, e.g., due to differences in average salaries. Thus, following common practice in economics (21), we use the purchasing power parity (PPP) in each country as an exchange rate to convert the value of 1 GB in their local currency to their equivalent value in USD, thereby reflecting the difference in the standard of living between countries. Fig. 1*A* summarizes the results of our experiment, where circles correspond to locations, colors represent average page load time, and diameters represent adjusted costs per Gigabyte. As can be seen, there is a clear digital inequality across the globe. The average page load time in some locations is four times longer than in others (about 47 vs. 12 s), and the cost per Gigabyte is orders of magnitude greater than in others ($43 vs. $0.08); *SI Appendix*, Table S1 for numeric values. Similar results were obtained when using direct conversion rates (*SI Appendix*, Fig. S1) and when using gross domestic product (GDP) in purchasing power parity (PPP) for each country (*SI Appendix*, Fig. S2).

To facilitate the comparison between the different locations, we plotted the distribution of the cost of one Gigabyte per location (Fig. 1*B*) as well as the distribution of the page load time per location, averaged over different webpages (Fig. 1*C*). Indeed, these distributions highlight the inequality between the locations. Moreover, to understand how the webpages themselves differ in terms of their complexity, we plotted the distribution of the page load time (s) per webpage, averaged over different locations (Fig. 1*D*). As can be seen, the page load time differs greatly across the webpages, ranging from 3.6 to 62.6, with the mean being 20.8. (Note that this is the time required to load the entire page).

Since the hardware specifications can affect the page load time, all measurements were taken using the same phone model, ensuring that the specifications were unified across locations and webpages. A low-end phone—Xiaomi Redmi Go—was used to help us understand the web-browsing experience of disadvantaged users; *SI Appendix*, Table S2 for the technical specifications of this phone. Note, however, that users with high income may afford high-end phones instead, which would make the inequality even greater. We found that the page load time and the cost per Gigabyte are not related to population size. Furthermore, when comparing capital to noncapital cities, we found the page load time to be almost identical and the cost per Gigabyte to be twice as high in capital cities. Interestingly, we found a positive correlation (*r*= 0.46, *P*= 0.0004, *SI Appendix*, Fig. S3) between page load time and cost per Gigabyte, indicating that those with poorer connection quality pay more, not less, than their counterparts.

### JavaScript Impact on Digital Inequality.

Arguably, digital inequality can be eliminated by providing cheap, fast connections worldwide. Unfortunately, this would not only take years to accomplish but would also be extremely costly, e.g., achieving universal, affordable, and good quality Internet access in Africa by 2030 would require 100 billion US dollars (22). A significantly cheaper alternative would be to make the webpages themselves “lighter,” by reducing their bandwidth and processing requirements. Such a solution would be desirable even if the lighter versions were slightly different from the original pages, as long as the compromise to the user experience is minimal. However, given the myriad webpages in the WWW, it may seem infeasible to analyze them all to identify the elements that are costly (in terms of bandwidth and processing time) and nonessential to the webpage (in terms of appearance and functionality).

Our key insight is to focus on JavaScript elements, which are not only computationally intensive but are also widely used across the WWW (23). Processing these elements is more demanding for web browsers than equivalently sized web components (24). Moreover, the download size of these elements often represents a considerable percentage of the total download size per page (25, 26). Surprisingly, despite its ubiquity, the cost of JavaScript processing on page load time is not fully understood to date. Motivated by this observation, we went 6 y back in time to understand how the processing of JavaScript affected the web-browsing experience on high-end vs. low-end phones over the years. To this end, we considered the 100 webpages most frequently visited in 2019. For each page, we retrieved a version per year over the period 2015 to 2020 from the Internet Archive Wayback Machine (27). The pages whose versions had technical issues were filtered out, ending up with a total of 55 webpages. We cloned the retrieved versions on our own web server and ran all experiments locally on that same server. This ensures that, when comparing webpages across different phones and years, we eliminate any differences related to network connectivity, access, and servers. For each year in 2015 to 2020, two mobile phones released in that year were used—a low-end phone and a high-end phone—to access the webpages retrieved in that year. We set up WebPageTest (19) to record the JavaScript processing time while accessing the pages from the different phones.

**Fig. 2.**
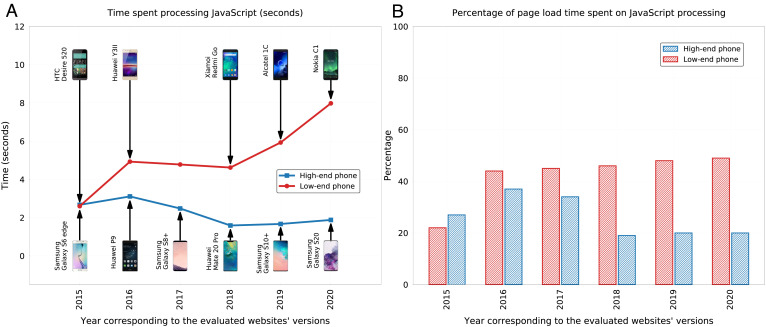
JavaScript processing time, measured on high-end vs. low-end mobile devices over the past 6 y. For each of the 100 webpages most frequently visited in 2019, we retrieved a version per year from 2015 to 2020. The pages whose versions demonstrated technical issues were filtered out, ending up with a total of 55 webpages. For every year in 2015 to 2020, two mobile phones released in that year were used—a high-end phone and a low-end phone—to access the webpages retrieved in that year; the phone models are specified in the figure. (*A*) Average JavaScript processing time (in seconds), measured using a high-end phone (blue curve) and a low-end phone (red curve). The data point for the low-end phone of 2017 was interpolated since no such phone was available to purchase at the time of the study. (*B*) Percentage of page load time spent on JavaScript processing, using a high-end phone (blue bar) and a low-end phone (red bar).

Fig. 2*A* shows the average time taken over the 55 webpages per year, using high-end phones (blue curve) and low-end phones (red curve); the phone models are named in the figure itself, and their technical specifications are provided in *SI Appendix*, Tables S3 and S4. As can be seen, the time spent processing JavaScript has decreased slightly on high-end phones, yet increased significantly on low-end phones over the years (from just over 2 s to nearly 8 s). Note that the increase is not due to a reduction in the processing power of the low-end phones used in our experiment; *SI Appendix*, Table S3. This suggests that the observed increase is attributed to the webpages becoming more computationally intensive over the years. It also suggests that popular webpages are designed with high processing power in mind, neglecting the less fortunate users who can afford only low-end phones, thereby exacerbating the digital inequality. Finally, Fig. 2*B* shows the percentage of page load time spent on JavaScript processing. As can be seen, in the past 3 y, the percentage was 20% for high-end phones and nearly 50% for low-end phones.

### Our Solution: Lite-Web.

So far, we demonstrated that a significant percentage of page load time is spent on JavaScript processing, and this percentage is greater for users of low-end phones. With this in mind, we propose a solution called Lite-Web, which focuses on producing lighter versions of webpages by optimizing the usage of JavaScript. Lite-Web is a hybrid approach, combining three of our state-of-the-art solutions, namely: SlimWeb (28) and JSCleaner (29), both of which block nonessential JavaScript elements, and Muzeel (30), which optimizes essential JavaScript elements. Let us now provide a basic description of these three solutions. For more details on each solution, *SI Appendix*, Notes 2, 3, and 4, and for an overview of related works, *Discussion* section.

SlimWeb is based on the idea that JavaScript elements can be classified based on their code, rather than their serving domains, as is the case with alternative commercial solutions. Relying on JavaScript code is particularly challenging since the code tends to span thousands of lines and may include obfuscated code (which is deliberately made difficult to understand to prevent reverse engineering), machine-generated code (which is often not human readable), or “uglified” code (which is generated via techniques that reduce code size at the expense of readability). By leveraging machine learning techniques, SlimWeb is not only capable of overcoming the above challenges but also classifying previously unseen elements, including unknown libraries, unidentified serving domains, and obfuscated code, all of which are commonly found in today’s Web. Such classification would not be possible using standard profiling techniques. As for the classes used in SlimWeb, they are based on the main JavaScript categories identified by experts in the web community (31). Of these classes, SlimWeb blocks the following three: 1) Advertising, which facilitates advertisement; 2) Analytic, which collects data about the users; and 3) Social, which enables social interactions such as likes and shares.

Having described SlimWeb, let us now move on to JSCleaner—the second component of our hybrid approach. Specifically, this rule-based solution is used to identify and block nonessential JavaScript elements that do not fall under any of the three classes used by SlimWeb. These elements are classified by JSCleaner as noncritical to the user experience if their code does not contain any functions that handle the page content or functionality.

Finally, let us describe the third component of our hybrid approach, namely Muzeel. Unlike the previous two solutions, which block nonessential JavaScript elements, Muzeel optimizes the code of essential elements. This is done by identifying and eliminating dead code—parts of the JavaScript code that are never used by the webpage. One of the reasons behind the existence of such code is the use of general-purpose libraries that provide far more functionalities—and hence far more code—than what is actually required by the page. The use of such libraries is a common practice among web developers to speed up the development process, with libraries such as jQuery appearing in 83% of mobile pages worldwide (32). The identification of dead code is challenging for several technical reasons stemming from the dynamic nature of the JavaScript programming language; see the works by Chugh et al. (33) and Obbink et al. (34) for more details. Muzeel utilizes a novel, interaction bot that emulates how a user may interact with the page. Such an approach enables the identification of JavaScript functions that can safely be removed without affecting the user experience and the overall page content.

### Evaluating Lite-Web.

To evaluate the impact of Lite-Web, we needed to run field experiments that are true to the web-browsing experience in developing regions. As a first step, it was crucial to identify a location where the inhabitants’ well-being is severely affected by poor Internet connectivity. Moreover, both the websites and the mobile phones used in the experiment needed to be popular in the identified location. Finally, the participants involved in the evaluation were required to be digital natives, who regularly browse the Internet and are familiar with the local network conditions.

Against these desiderata, we chose the Gilgit-Baltistan province in Pakistan, where poor Internet quality causes severe disruption to students, preventing them from keeping up with their peers. This was demonstrated by the students’ protests in July 2020 demanding digital rights (35), leading to the hashtag #Internet4GilgitBaltistan becoming the second-highest ranked on Twitter in Pakistan (36). As for the mobile phone on which the experiments are conducted, we chose the same low-end phone used earlier, namely QMobile i6i 2020, since it is manufactured by a popular Pakistani company. Finally, we used the Tranco-list (37) to retrieve the 100 Pakistani webpages that were most frequently visited in 2021. Now, we are ready to evaluate the impact of Lite-Web both quantitatively (using automated measurements) and qualitatively (through a user study).

### Quantitative Evaluation.

We sent two teams to four different locations within the Gilgit-Baltistan to measure the impact of Lite-Web based on four evaluation metrics: page load time, Speed Index, page size, and JavaScript processing time. More specifically, the four locations are Taus, Hundur, Sherqilla, and Puniyal, all marked on the map in *SI Appendix*, Fig. 4. The measurements were conducted using the WebPageTest framework (19), where the QMobile i6i mobile phone was controlled through a laptop to automatically launch both the original and the Lite-Web versions of each of the 100 Pakistani webpages. This experiment was repeated three times to account for any subtle variations that may arise when the same webpage is visited multiple times. As a result, we ended up with a total of 1,200 visits (4 locations × 100 webpages × 3 visits).

**Fig. 3.**
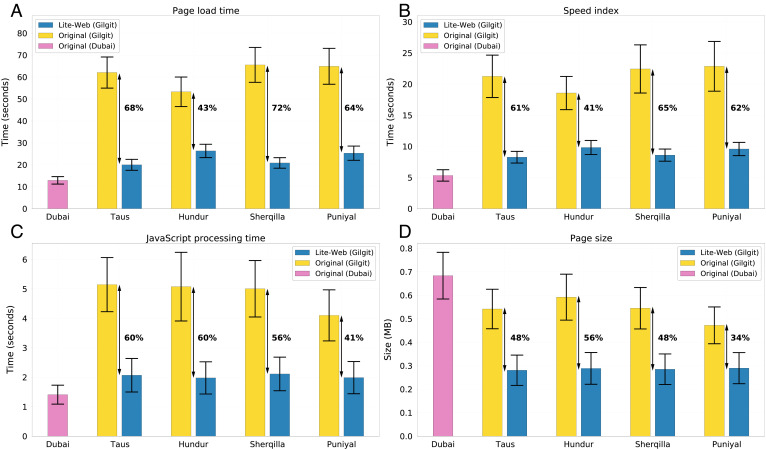
Quantitative evaluation of Lite-Web. Using the 100 most frequently visited Pakistani webpages in 2021 to evaluate Lite-Web in four locations situated in the Gilgit-Baltistan province—namely Taus, Hundur, Sherqilla, and Puniyal. The evaluation is done by comparing the Lite-Web version (blue bar) to the original version (yellow bar) on the same low-end phone (QMobile i6i 2020) under the same cellular network conditions (SCOM 4G). Additionally, both the original and the Lite-Web versions are compared to a baseline (pink bar), whereby the same 100 webpages are running on a high-end phone (Samsung Galaxy S20+ 2020) under a cellular network in Dubai (Etisalat 4G+). Error bars represent the 95% confidence intervals. (*A*) Evaluating page load time. (*B*) Evaluating Speed Index. (*C*) Evaluating JavaScript processing time. (*D*) Evaluating page size.

Fig. 3*A* depicts the impact of Lite-Web on page load time—a measure representing the elapsed time from initiation (when the user types in the Web address) to completion (when the page is fully loaded). As shown in the figure, the reduction in page load time across the four locations is 68% (in Taus), 43% (Hundur), 72% (Sherqilla), and 64% (Puniyal), with the average time reduced from 61 to 23 s. To determine whether this improvement is sufficient to bridge the digital divide, we compared Lite-Web’s outcome to what the people of Gilgit-Baltistan would experience if they were browsing the same 100 Pakistani pages in a developed region (Dubai) on a high-end phone (Samsung Galaxy S20+) using a superior cellular network connection (4G+). As can be seen in Fig. 3*A*, the additional waiting time that users in Gilgit-Baltistan would suffer compared to their privileged counterparts is reduced from 48 s (average difference between yellow bars and the pink bar) to just 10 s (average difference between blue bars and the pink bar), amounting to an overall reduction of about 80%.

Fig. 3*B* corresponds to the second performance metric, namely Speed Index, which measures the time taken for the contents of a page to be visibly populated and displayed to the user. Again, the use of Lite-Web results in a significant improvement across all four locations, reducing the gap between developed and developing regions by about 70%. Fig. 3*C* depicts the impact of Lite-Web on the time spent processing JavaScript. As can be seen, the time drops by an average of 54% across locations, and the gap between Gilgit-Baltistan and Dubai drops by about 80%. Fig. 3*D* shows how the size of different webpages is reduced by Lite-Web. Specifically, the page size averaged across webpages, and locations is reduced by about 50% (from 0.54 to 0.28 MB). Notice that the average page size in Gilgit-Baltistan (without Lite-Web’s improvements) is slightly smaller than Dubai’s. This is because high-end phones request bigger-size images compared to low-end alternatives. However, after using Lite-Web, the webpages become smaller than those downloaded in Dubai by about 60%.

Finally, we evaluated the impact of each of Lite-Web’s constituent parts, namely SlimWeb, Muzeel, and JSCleaner. As shown in *SI Appendix*, Fig. S5, SlimWeb is the most impactful in terms of the time-based metrics (page load time, Speed Index, and JavaScript processing time), while SlimWeb and Muzeel have a comparable impact in terms of page size reduction.

We compared Lite-Web to two state-of-the-art industry solutions that are widely deployed, namely Opera Mini (38) and Brave (39). In particular, Opera Mini sends users’ webpage requests to their proxy server, where the pages are first requested and then compressed before being sent back to the user in order to reduce the transfer size and speed up the browsing experience. It is estimated that Opera Mini has about 170 million users (40). However, Opera Mini is prone to breaking interactive sites that rely heavily on JavaScript. Brave, on the other hand, is a privacy-focused browser, which automatically blocks online advertisements and website trackers in its default settings. As of December 2021, Brave has more than 50 million monthly active users and 15.5 million daily active users (41).

Similar to the evaluation done earlier, we wanted to compare Lite-Web to these two state-of-the-art industry solutions based on four evaluation metrics: page load time, Speed Index, page size, and JavaScript processing time. The measurements were conducted in the city of Lahore in Pakistan using the WebPageTest framework (19), which controlled the QMobile i6i mobile phone to automatically launch the Lite-Web, Opera Mini, and Brave versions for each of the 100 most popular Pakistani webpages. This experiment was repeated three times to account for any subtle variations that may arise when the same webpage is visited multiple times. Note that the results for Opera Mini are depicted only for the page load time and the Speed Index, since the WebPageTest framework was unable to collect the remaining two evaluation metrics. The results of this evaluation are depicted in *SI Appendix*, Fig. S6. As can be seen, Lite-Web achieves improvements ranging between 24% and 57% depending on the benchmark and the evaluation metric.

### Qualitative Evaluation.

To assess whether the above improvements come at the expense of the page look or functionality, we recruited 200 students from two high schools in the Gilgit-Baltistan province. Those students were randomly assigned to control and treatment groups of equal sizes. After that, the 100 Pakistani webpages were assigned to the students as follows: The webpages were divided into 25 disjoint, exhaustive, and equally sized lists. Then, each list was assigned to four randomly chosen students from the control group (who interacted with the original versions of the webpages), as well as four randomly chosen students from the treatment group (who interacted with the Lite-web versions). All participants interacted with their assigned versions for 15 min using the same low-end phone model (QMobile i6i) equipped with a cellular data connection. Importantly, none of the participants knew the purpose of the study or the group to which they belonged. This was done to minimize the risk of subject bias, whereby participants tend to behave according to what they believe the experimenter wants to see. The study was conducted by a CITI-trained (42) person following Institutional Review Board (IRB) approval (HRPP-2021 to 2032) from New York University Abu Dhabi. Furthermore, a letter of approval was obtained from the school principal to conduct the study on the school premises. Social distancing measures were observed, and participants were asked to wear masks throughout the study; *SI Appendix*, Fig. S7. As a token of our appreciation, we donated twelve QMobile QTab v7 Pro tablets to the schools’ libraries to be used for educational purposes.

**Fig. 4.**
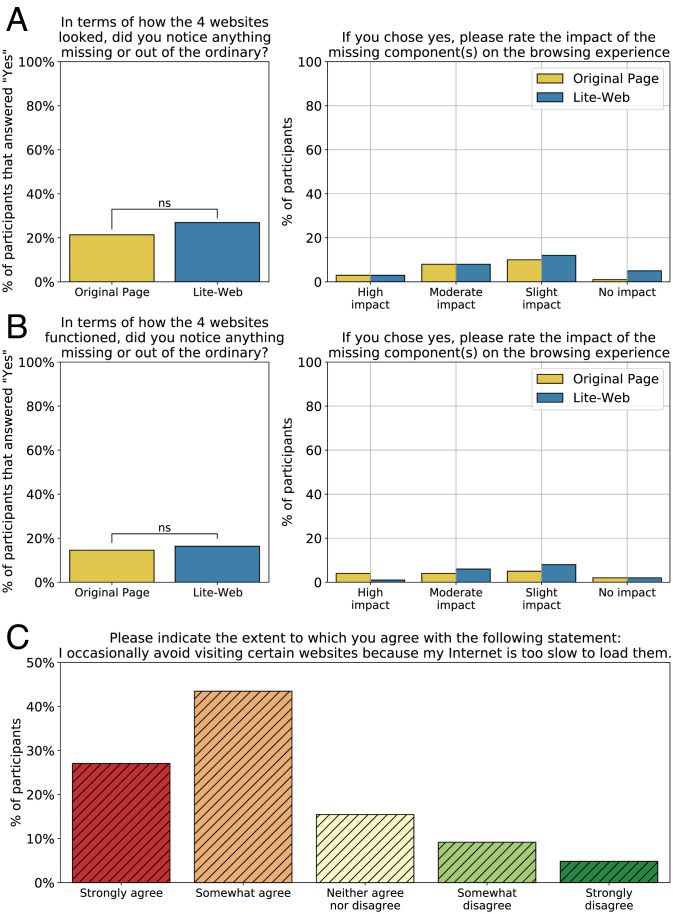
High school students’ evaluation of Lite-Web’s impact on the appearance and functionality of websites. Each participant interacted with 4 of the 100 Pakistani websites most frequently visited in 2021; the control and treatment groups interacted with the original and Lite-Web versions of these websites, respectively. (*A*) *Left* *panel*: Percentage of participants who answered “Yes” to the question: “In terms of how the four websites looked, did you notice anything missing or out of the ordinary?” (ns = not significant; *P* = 0.42); those who answered “Yes” were subsequently asked: “If you chose yes, please rate the impact of the missing component(s) on the browsing experience;” the distribution of their responses is depicted in the *Right* *panel*. (*B*) Similar to (*A*) but for questions asking about how the websites functioned, rather than how the websites looked (ns = not significant; *P* = 0.85). (*C*) Responses of all participants (control and treatment) to the question: “Please indicate the extent to which you agree with the following statement: I occasionally avoid visiting certain website because my Internet is too slow to load them.”

The results of the user study are summarized in Fig. 4. Specifically, the left panel of Fig. 4*A* summarizes the users’ evaluation of the webpages’ appearance. This shows no significant difference between the control and treatment groups. In other words, we found no evidence indicating that the performance gains attributed to Lite-Web come at the expense of appearance. Similar results were observed when accounting for the gender and age of participants. Fig. 4*A*, *Right* focuses on the users (in both the control and treatment) who noticed something missing in terms of appearance; those users were asked to assess the impact of the missing components on the browsing experience. As can be seen, the treatment looks very similar to the control, with the only difference being two additional participants (out of 100) who indicated a slight impact of the missing components and four additional participants who indicated no impact.

Fig. 4*B* is similar to Fig. 4*A* except that it evaluates the impact of Lite-Web on the webpages’ functionality rather than appearance. Again, the left panel shows no significant difference between the control and treatment. In other words, we found no evidence that Lite-Web’s performance gains come at cost to functionality. Accounting for users’ gender and age reveals similar trends. Fig. 4*B*, *Right* focuses on the few participants who noticed something missing in terms of functionality. Five additional users (out of 100) in the treatment group indicated a slight to moderate impact, and three additional users in the control group indicated a high impact. Finally, after participating in the study, all 200 students were asked to indicate the degree to which they agree with the following statement: “I occasionally avoid visiting certain websites because my Internet is too slow to load them.”

Fig. 4*C* depicts the distribution of the responses, showing that the majority (70%) agree (somewhat or strongly) with the statement. These findings suggest that students in the Gilgit-Baltistan province are excluded from certain webpages because of being on the less fortunate side of the digital divide. More broadly, these results suggest that people in developing regions are in need of solutions such as Lite-Web to empower them to reach otherwise practically unreachable parts of the World Wide Web. As a sensitivity analysis, we repeated the same experiment but with a few modifications. First, we divided the 100 websites based on deciles and randomly picked a website from each part, resulting in just 10 websites. Second, we recruited students from Lahore University of Management Sciences. Third, we recruited 800 participants and asked each of them to evaluate all 10 webpages, resulting in 800 evaluations per webpage. The evaluation yielded broadly similar results.

## Discussion

Our goal was to understand the extent of the digital divide phenomenon worldwide and propose a scalable and affordable solution that can potentially alleviate it. We measured the mobile data cost and page load time in 56 cities and found evidence of digital inequality across the globe. In particular, we found the cost of 1 GB in some locations to be orders of magnitude greater than in others and the average page load time to be four times as long. Crucially, in each location, the results were averaged over the same 100 webpages, and the measurements were taken using the same low-end phone model, to unify the experimental setup across locations. An interesting avenue for future work would be to scale up this experiment, covering more areas within countries and over time, to chart the digital divide. Another direction for future work is to extend Lite-Web such that it not only removes deadcode and blocks noncritical JavaScript files but also identifies and removes potentially malicious JavaScript code from existing webpages, thereby enhancing the users’ security.

In an attempt to identify a solution that can bridge the digital divide, we focused on JavaScript elements, which are more computationally intensive than any other equally sized web component. Specifically, we studied how the above 100 webpages have changed from 2015 to 2020 and found that the time spent processing JavaScript has remained largely the same on high-end phones but has increased significantly on low-end phones over the years. This suggests that webpages are designed with high processing power in mind while neglecting the less fortunate users who can afford only low-end phones, thereby exacerbating the digital inequality. More importantly, we found that a significant percentage of page load time is spent on JavaScript processing, and this percentage is greater for users of low-end phones.

Motivated by this key observation, we proposed a solution called Lite-Web, consisting of three algorithms designed specifically to optimize the usage of JavaScript elements in today’s web. We evaluated Lite-Web across four locations in a province of Pakistan known for its poor Internet connectivity, namely Gilgit-Baltistan. The evaluation focused on the 100 most popular Pakistani pages and was done using a locally manufactured low-end phone. This demonstrated Lite-Web’s ability to substantially reduce the size and loading time of webpages, thereby effectively transforming the local browsing experience to that of Dubai’s residents who can afford flagship phones with fast Internet connections.

Based on user studies conducted at two high schools and a university in the region, we found no evidence that the performance gains obtained by Lite-Web come at the expense of the look and functionality of the webpages. However, given that Lite-Web blocks ads, it can reduce the revenue of the content providers and may disadvantage companies in developing regions as they can no longer advertise their services to the users. Having said that, it should be noted that Lite-Web is not the only solution that blocks ads and analytics. In fact, one of the main features of the “Brave Browser” (39)—a very successful modern browser with more than 50 million monthly active users and 15.5 million daily active users—is to block ads and trackers. Moreover, ads constitute only one of the categories blocked by SlimWeb, which in turn constitutes only one of three components of Lite-Web. If need be, the ad-blocking feature of SlimWeb can be disabled, in which case the solution would still provide significant speedups to the page load time (28).

### Limitations.

Our study comes with a number of limitations. First, when reporting the page load time and mobile data cost across cities (Fig. 1), our data represents a single point-in-time snapshot of performance and price. Mobile network performance evolves rapidly, both due to network upgrades as well as increased usage of infrastructure, but these factors are not considered in our analysis. Similarly, we do not consider the role of policy and competitive factors that drive the data cost. Moreover, although participants were instructed to purchase a plan they considered to be affordable, this plan is not representative of the entire spectrum of plans available in their respective city. Having said that, our experiment facilitates a comparison across cities since the price was deemed affordable by an undergraduate student who came from that city (in addition to the experimental protocol which controlled for processing power, web browser, pages visited, connection medium, and time of day). As such, the analysis in Fig. 1 provides evidence of digital inequality across cities but should not be interpreted beyond that.

### Related Work.

Over the past decade, expanding Internet access has become a target for international advocacy efforts from the United Nations, and many solutions have been proposed to provide affordable, high-quality connection to everyone. However, such efforts rely on critical infrastructure that would require years to build and hundreds of billions of US dollars to fund (43). A significantly cheaper alternative is to make the webpages lighter for developing regions. Surprisingly, this alternative has only just started gaining attention. For example, Facebook has introduced a solution called Facebook Lite (44) for Android users with limited connectivity and low-end phones. However, this solution is designed solely for Facebook. Another initiative is Google’s Accelerated Mobile Pages (AMP) (45), which provides a framework that can assist web developers in creating lighter versions of their webpages. Unfortunately, AMP does not consider existing webpages but rather requires the creation of new ones from scratch. This makes it hard to deploy on a massive scale, especially given the billions of webpages already present in the WWW.

From the developer’s perspective, one way to reduce the size of JavaScript files before they are embedded into the page is to use uglifiers (46, 47). These rely on removing nonessential characters such as white spaces and newlines from JavaScript files to improve transmission efficiency. However, unlike our Lite-Web solution, uglifiers do not reduce JavaScript processing time—a major contributor to the digital inequality, as our experiments have shown. From the user’s perspective, several JavaScript blocking tools (48–52) can be used to reduce the amount of JavaScript transferred to their browsers. However, these tools are restricted to a predetermined blocklist and are not equipped with any sort of intelligence that can automatically classify previously unseen JavaScript elements to determine whether they should be blocked. A very recent solution called Percival (53) has shown promising results in blocking ads using deep learning. It intercepts images obtained during page execution to flag potential ads. However, this solution is computationally intensive, resulting in a nonnegligible performance overhead on desktop PCs. As such, it cannot be applied on low-end mobile phones with limited computational power.

In a recent work (54), the authors proposed WebMedic—a method to remove less-useful (rather than entirely unused) functions from the page. They found that 20% of the memory can be saved for the majority of webpages while preserving 80% of the functionality. However, further research is needed to maximize the speedup while minimizing the impact on the page functionality. Other ways to improve the browsing experience are offered by platform-based solutions. For instance, Apple News (55) is a news aggregator app developed exclusively for Apple mobile devices, whereas Instant Articles (44) is a tool that allows publishers to create fast and interactive content on Facebook. However, such solutions are narrow in scope and are not generalized to all devices and/or all webpages.

### Conclusions.

We saw how the gap between high-end and low-end phones has increased over the past 5 y in terms of JavaScript processing. If this trend continues without any interventions, it would lead to a segregation of disadvantaged and advantaged users, whereby the former are practically unable to access the webpages that cater to the latter. Such segregation would violate the net neutrality principle (56), which requires treating all Internet traffic equally, without discriminating or charging differently based on user, content, website, location, type of equipment, or access medium. Our findings call for attention from researchers and policymakers alike to mitigate disparity and adhere to the net neutrality principle across the globe. More broadly, Internet connectivity has arguably become a basic human right in the twenty-first century, and the emerging literature on reducing web complexity (34, 39, 44, 45, 54, 57–63) constitutes a promising step toward realizing the United Nation’s vision “to ensure that digital technologies are built on a foundation of respect for human rights and provide a meaningful opportunity for all people and nations” (16).

matseccnt1

## Methods

Our proposed Lite-Web solution combines three algorithms that we developed to reduce the processing cost of JavaScript in today’s webpages, namely SlimWeb (64), JSCleaner (29), and Muzeel (30). For a given webpage, Lite-Web first runs SlimWeb’s machine learning classifier to identify and block JavaScript elements that are nonessential to the user experience; *SI Appendix*, Note 2 for more details. A user study (64) showed that, in order to achieve faster browsing, people are willing to sacrifice parts of the page that are responsible for: 1) advertising, 2) analytics, and 3) social interactions. Based on this finding, all JavaScript elements belonging to the above three categories are blocked by Lite-Web. Additionally, Lite-Web runs a modified version of the rule-based classification used by JSCleaner to identify and block JavaScript elements that are noncritical to the page content or interactive functionality. More details on how Lite-Web modifies JSCleaner’s rules can be found in *SI Appendix*, Note 3.

So far, Lite-Web preserves JavaScript elements that are identified as essential by SlimWeb and the modified JSCleaner rules. By analyzing these preserved elements, we found many of them to be large JavaScript libraries that are incorporated wholly into the page, even though only a few functions of these libraries are utilized (30). This key observation suggests that optimizing webpages can go beyond eliminating nonessential JavaScript elements, by optimizing the essential ones. This optimization is done through the elimination of functions that are included in the essential elements yet not used by the webpages. This is precisely what Muzeel is designed to do. The elimination of unused functions provides data cost savings (since the files containing such functions are often quite large) as well as performance improvements (60) (since the number of functions that require processing is now reduced). Further details on how Muzeel operates can be found in *SI Appendix*, Note 4.

When evaluating Lite-Web in Gilgit-Baltistan, we deployed Lite-Web in a cloud server hosted in Pakistan. This server maintained a database of JavaScript elements extracted from the 100 most popular Pakistani webpages, labeled by SlimWeb and JSCleaner as either essential or nonessential. The server caches a modified version of the essential ones, which is stripped out of any unused functions by Muzeel. The phones’ browsers were configured to utilize our Lite-Web server as a web proxy. As such, JavaScript requests are either deemed nonessential by the proxy and subsequently blocked, or deemed essential, in which case the Muzeel’ed versions of these elements are sent back from the server cache. All other web elements’ requests, apart from JavaScript, are served live from the Internet.

## Supplementary Material

Appendix 01 (PDF)

## Data Availability

Our data were collected from several experiments that we ran: a) in the wild page load times and cost collected from 56 cities around the world, b) in lab experiments on JavaScript processing times over past 6 y, and c) in the wild quantitative evaluation of Lite-Web from two schools in Pakistan. The whole data will be shared upon publication under the following repository https://github.com/comnetsAD/digital-divide.
