# Supplementary material for: Towards a World Wide Web without digital inequality
Source: Proc Natl Acad Sci U S A. 2023 Jan 9;120(3):e2212649120. doi: 10.1073/pnas.2212649120 (PMC9934074; doi:10.1073/pnas.2212649120)
Supplement: Supplementary file 1 — Appendix 01 (PDF) [file pnas.2212649120.sapp.pdf]

Supporting Information for

Towards bridging the digital divide in developing regions

Moumena Chaqfeh, Rohail Asim, Bedoor AlShebli, Muhammad Fareed Zaffar, Talal Rahwan, Yasir Zaki

Corresponding Yasir Zaki.
E-mail: yasir.zaki@nyu.edu

This PDF file includes:

Supporting text
Figs. S1 to S8
Tables S1 to S5
SI References

Supporting Information Text

Supplementary Note 1: Feature selection. The objective of this supplementary note is to summarize the feature selection for both SlimWeb and JSCleaner; for more details see (1) and (2). To represent each JavaScript element embedded in a webpage as a vector of features, we utilized the set of 1262 features proposed in (2). These features represent all Application Programming Interfaces (APIs) that facilitate interacting with the Document Object Model (DOM) of HTML, which is the main programming interface that defines the logical structure of HTML documents and the way they can be accessed and manipulated (3, 4), including properties, methods, and event handlers. By knowing all the APIs that a certain JavaScript element uses, we can infer the purpose of that element, since those APIs cover all the ways in which JavaScript can interact with the webpage (including reading, writing, and event-handling).

To design a complete set of features, the full list of the aforementioned APIs along with their properties, methods, and events are considered. Each feature was manually labeled according to the DOM (3) and the DOM HTML APIs specifications (4), with the objective of categorizing each of them to aid the rule-based classification in JSCleaner. It is worth noting that only the features, without their labels, are required for the machine learning classification in SlimWeb. Four possible labels were identified:

1. **Read feature:** A feature that can only be used to extract information from webpages.
2. **Read/Write feature:** A feature that can be used to read from or write to webpages.
3. **Write feature:** A feature that can only be used to modify a given page, by creating or adding new web elements to the page, or by changing existing elements in the page. For example, the "appendChild" method of the Node interface writes to the page by adding a child node to an existing parent node.
4. **Event feature:** A feature that handles a user interactivity event (such as a click) or a web event (such as "DOMContentLoaded" which fires when the initial document is completely loaded and parsed).

Duplicate features (properties or methods that are found in more than one API, such as id, name, type, width, length, and height) were removed from the final set of features due to their common utilization by the JavaScript language core (5). This resulted in a final set of 1,262 features, each with a unique label, which can be reproduced by extracting all the APIs specified in (3) and (4), along with their properties, methods, and events. The final set is used for the rule-based classification of JSCleaner. On the other hand, when using SlimWeb, we created a reduced set of 508 features for a faster on-the-fly classification for SlimWeb; this was done using recursive feature elimination with cross-validation.

Supplementary Note 2: SlimWeb. The objective of this supplementary note is to summarize the design details of SlimWeb; see (1) for further details. SlimWeb is built around a machine learning algorithm that classifies each JavaScript element embedded in a given webpage into one of eight categories. These categories are based on the best web practices defined by experts in the field (6). Specifically, the eight categories are as follows:

1. **Advertising:** JavaScript elements related to advertising and marketing.
2. **Analytics:** JavaScript elements that measure or track users by recording their actions.
3. **Social:** JavaScript elements that enable social features (such a sharing) in webpages.
4. **Video:** JavaScript elements that enable video players and manage streaming functionality.
5. **Utilities:** JavaScript elements related to developer utilities, such as API clients, site monitoring utilities, and fraud detectors.
6. **Hosting:** JavaScript elements brought by web hosting platforms (such as WordPress, Wix and Squarespace), which include publicly hosted open source libraries (such as jQuery) served over different public CDNs (Content Delivery Networks) and private CDN usage.
7. **Customer success:** JavaScript libraries brought from customer support/marketing providers that offer chat and contact solutions.
8. **Content:** JavaScript libraries brought by content providers or publishing-specific affiliate tracking, including tag management elements that tend to load other JavaScript elements and initiate specific tasks.

To train different supervised learning models, a labeled JavaScript dataset was created by crawling 20,000 popular pages, and extracting the JavaScript elements of those pages. The domain names behind these elements were cross-checked against a publicly available HTTP Archive repository (7) that lists existing JavaScript libraries along with their domain names, and their respective category from the above list. This process yielded 127,000 matches, each corresponding to a labeled (i.e., categorized) JavaScript element. This constitute SlimWeb's training set, in which each JavaScript element is represented by a vector of features, taken from Supplementary Note , as well as a label taken from the above eight categories.

Using the training set, we experimented with six supervised learning models. These consisted of three distance-based classifiers: K-Nearest Neighbors (KNN), Support Vector Machine (SVM) and Linear Support Vector Classifier (LSVC)); two tree-based classifiers: Random Forest Classifier (RFC) and XG Boost; and a simple neural network model. Next, we specify the parameterization used for each model.

Starting with distance-based classifiers, for KNN we chose the output class to be the maximum class represented in the K-nearest neighbors. We chose the value $K = 293$ since it gave the best classification performance on the training set (the evaluation metrics are described later on in this section). For SVM and LSVC, we used the standard training methodology over the entire feature space.

Out of the available tree-based classifiers, RFC was selected due to its ability to reduce over-fitting and improve accuracy. We used the Exhaustive Grid Search approach to adjust the hyper-parameters. The number of trees was set to 250 as this yielded the best accuracy. As for XG Boost, it was selected due to its superior performance in several machine learning challenges (8). We set the maximum depth to 32, since larger values did not increase the accuracy. The learning rate was set to 0.3.

Finally, we experimented with a simple neural network, and used two hidden layers since the accuracy did not improve with a greater number of such layers. The *ReLU* (Rectified Linear Unit) (9) activation function was used in the hidden layers, while the output layers utilized the *softmax* activation function. When evaluating the above models, we used the following standard performance metrics:

- Recall, which is computed as follows:

$$Recall = \frac{\text{True Positives}}{\text{True Positives} + \text{False Negatives}} \quad [1]$$

- Precision, which is computed as follows:

$$Precision = \frac{\text{True Positives}}{\text{True Positives} + \text{False Positives}} \quad [2]$$

- F1-Score, which measures the classification accuracy by combining both the precision and recall, and is computed as follows:

$$F1\text{-}score = \frac{2 \times Precision \times Recall}{Precision + Recall} \quad [3]$$

Supplementary Table S5 presents a summary of the evaluation results for the different supervised learning models. Each value in the table represents an overall average of the corresponding metric, which is computed across all the eight different categories. The detailed evaluation of the models for each category is shown in Supplementary Fig. S8. Based on these results, we opted to use the simple neural network, as it outperformed all other alternatives.

Based on the above evaluation, Lite-Web uses a simple neural network to classify JavaScript elements into one of the eight categories outlined earlier. Then, as mentioned in the main manuscript, Lite-Web blocks the elements that are classified as *Advertising*, *Analytics*, or *Social*.

Supplementary Note 3: JSCleaner. JSCleaner employs a rule-based classification using the labeled features described in Supplementary Note to classify JavaScript elements into essential and non-essential. In Lite-Web, we modified the original version of JSCleaner which considers a third class that is referred to as replaceable JavaScript. Instead of replacing these elements with HTML as in the original JSCleaner, we consider them as essential. Using the 1,262 features described earlier, JavaScript elements are labeled by the modified version of JSCleaner according to the following rules:

1. **Essential classification rule:** If a JavaScript element contains a set of event features or writing features then it is classified as *essential*.

2. **Non-essential classification rule:** If a JavaScript element neither contains event features nor writing features then it is classified as *nonessential*.

Supplementary Note 4: Muzeel. The objective of this supplementary note is to summarize the design details of Muzeel; see (10) for further details. Muzeel automatically identifies the order of events needed to cover all possible states a webpage can reach at run-time. Based on this, it dynamically analyzes JavaScript elements to identify and eliminate their unused functions, i.e., their dead-code. This is done by considering both the page-load events and the user interactivity events of a given page. Such events are handled by “event handlers” which call specific JavaScript functions whenever events are fired. While existing approaches (11) identify only the JavaScript functions that are required for the page load events, Muzeel additionally identifies the JavaScript functions required by user events through user-interactivity emulation. Below is a detailed description of the three main phases of Muzeel, which are: pre-processing, dead-code identification, and dead-code elimination.

Pre-processing: During this phase, Muzeel modifies the functions from all JavaScript elements used in the page. This modification ensures that, whenever a function is called by an event, it outputs a unique ID to the browser’s console. This way, if a function’s ID was not logged to the browser’s console, it indicates that this function is never used by the page and thus can be safely removed.

Dead-code identification: Muzeel loads a webpage in an automated browser to emulate the user interactivity events once the page has loaded. This way, JavaScript functions that handle page load events or user interactivity events can be logged to the browser console. A user interactivity event is associated with a *page element*, defined as an object that appears in an HTML tag within the Document Object Model (DOM) of the page (such as image, button, or navigation element). Muzeel identifies the page elements and extracts the set of all user events associated with each of them to drive the user interactivity emulation. To this end, it uses `XPaths` to uniquely identify page elements across reloads. To emulate user interactivity on a webpage, Muzeel triggers all user events using a browser automation tool. Here, the dependencies between events should be taken into consideration. For instance, some events are only successful if triggered after others. Similarly, triggering certain events may prevent the interactivity with other events. Muzeel considers such dependencies when emulating user interactivity. Further details on elements' identification and how Muzeel emulates the user interactivity can be found in (10).

Dead-code elimination: Using the browser's console logs—which contains the IDs of the JavaScript functions that were called by the page—Muzeel annotates the *used* functions. This way, functions that are never called can be removed from their respective JavaScript files. Muzeel implicitly considers nested functions that are called upon the execution of other functions due to user interactivity. Whenever a function handling a user interactivity event calls a set of nested functions, the IDs of all those functions are logged to the browser console. Additionally, the elimination of a given function leads to the elimination of all the nested ones. By following these steps, Muzeel obtains a complete trace of the used functions and an accurate identification of dead-code in a given webpage.

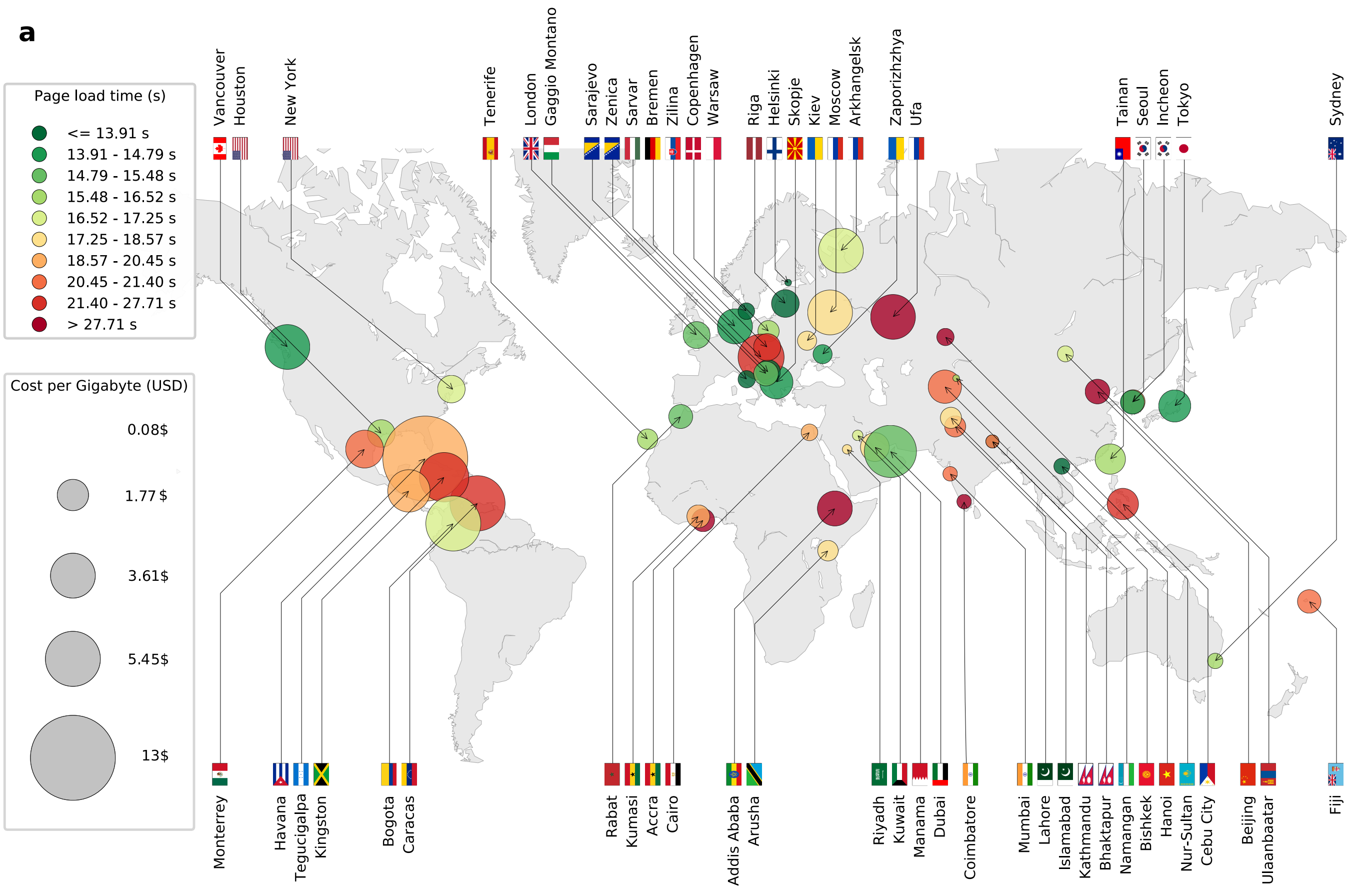

Fig. S1. Average page load time and data cost across different locations. Similar to Figure 1a in the main article, except that the cost per Gigabyte is computed based on direct conversion rate instead of using the purchasing power parity.

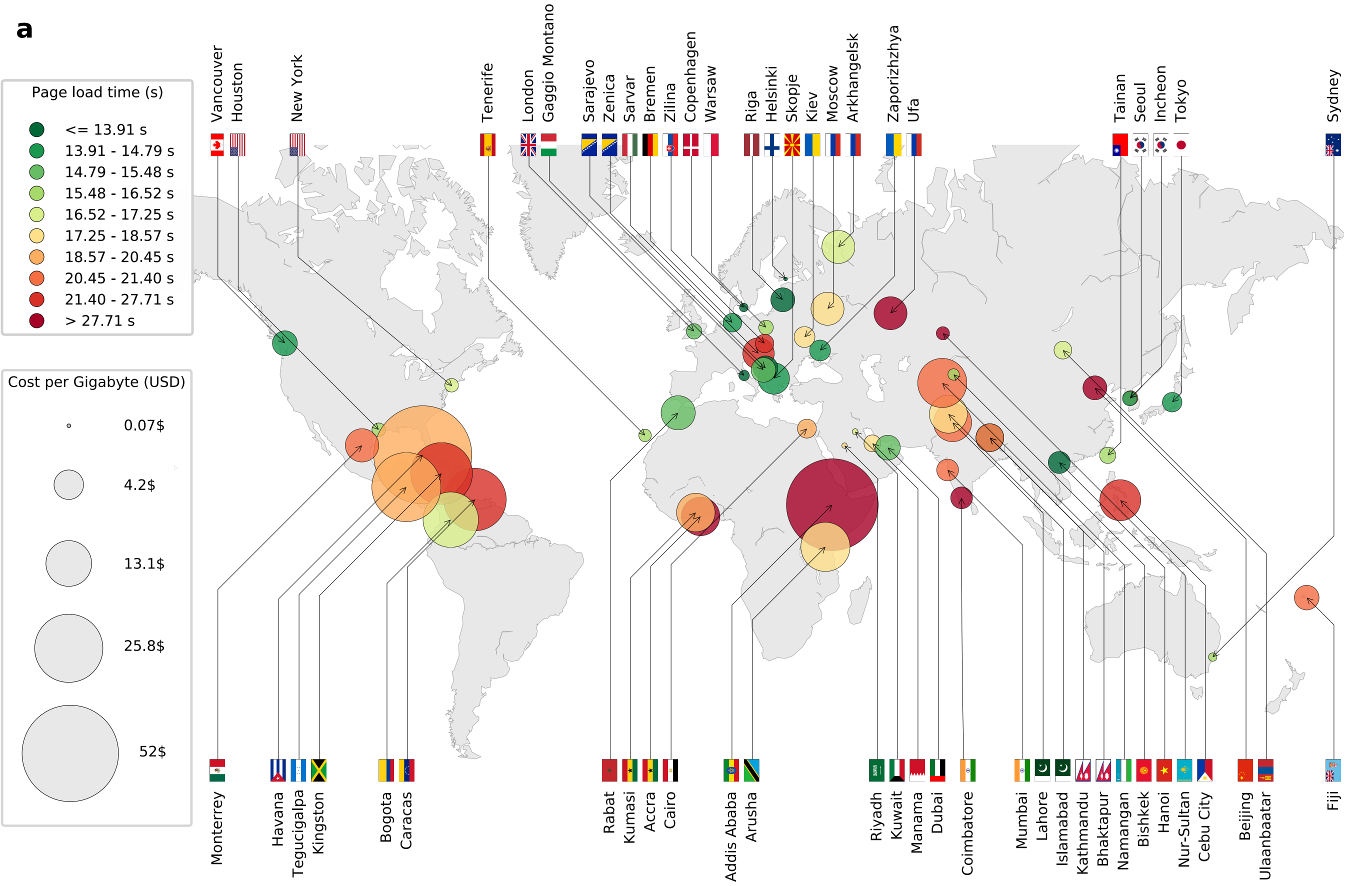

Fig. S2. Average page load time and data cost across different locations. Similar to Figure 1a in the main article, but instead of using the purchasing power parity (PPP), the cost per Gigabyte is computed based on the gross domestic product (GDP) in purchasing power parity (PPP) for each country.

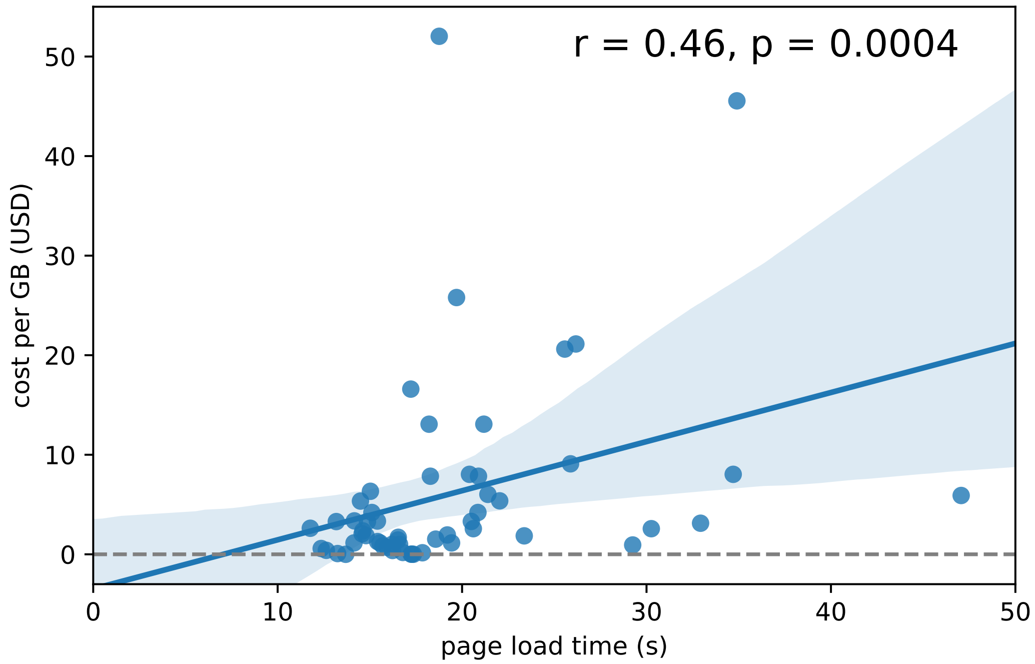

Fig. S3. Correlation between page load time and cost per Gigabyte. Each data point represents one of the 56 cities analyzed in Figure 1 of the main article.

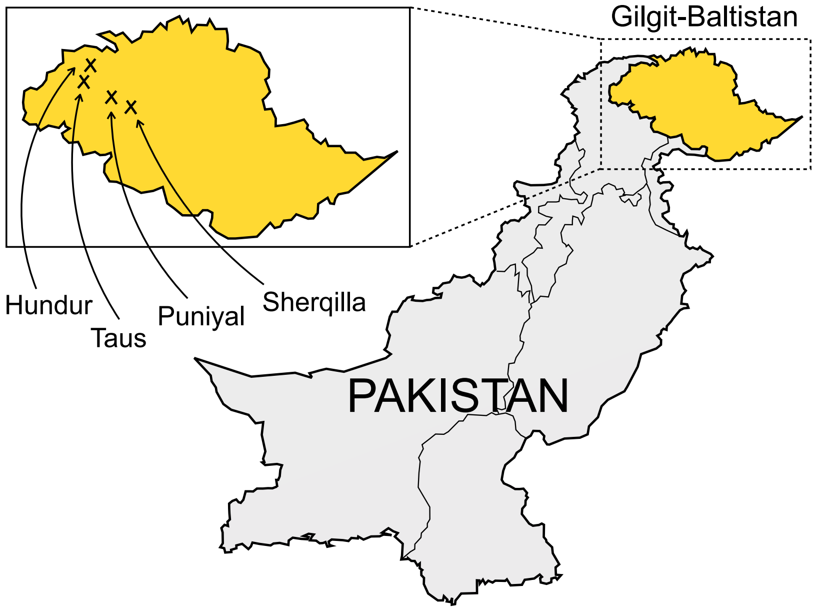

Fig. S4. Field experiment locations. The map of Pakistan, highlighting the Gilgit-Baltistan province, and the locations in which the field experiment took place.

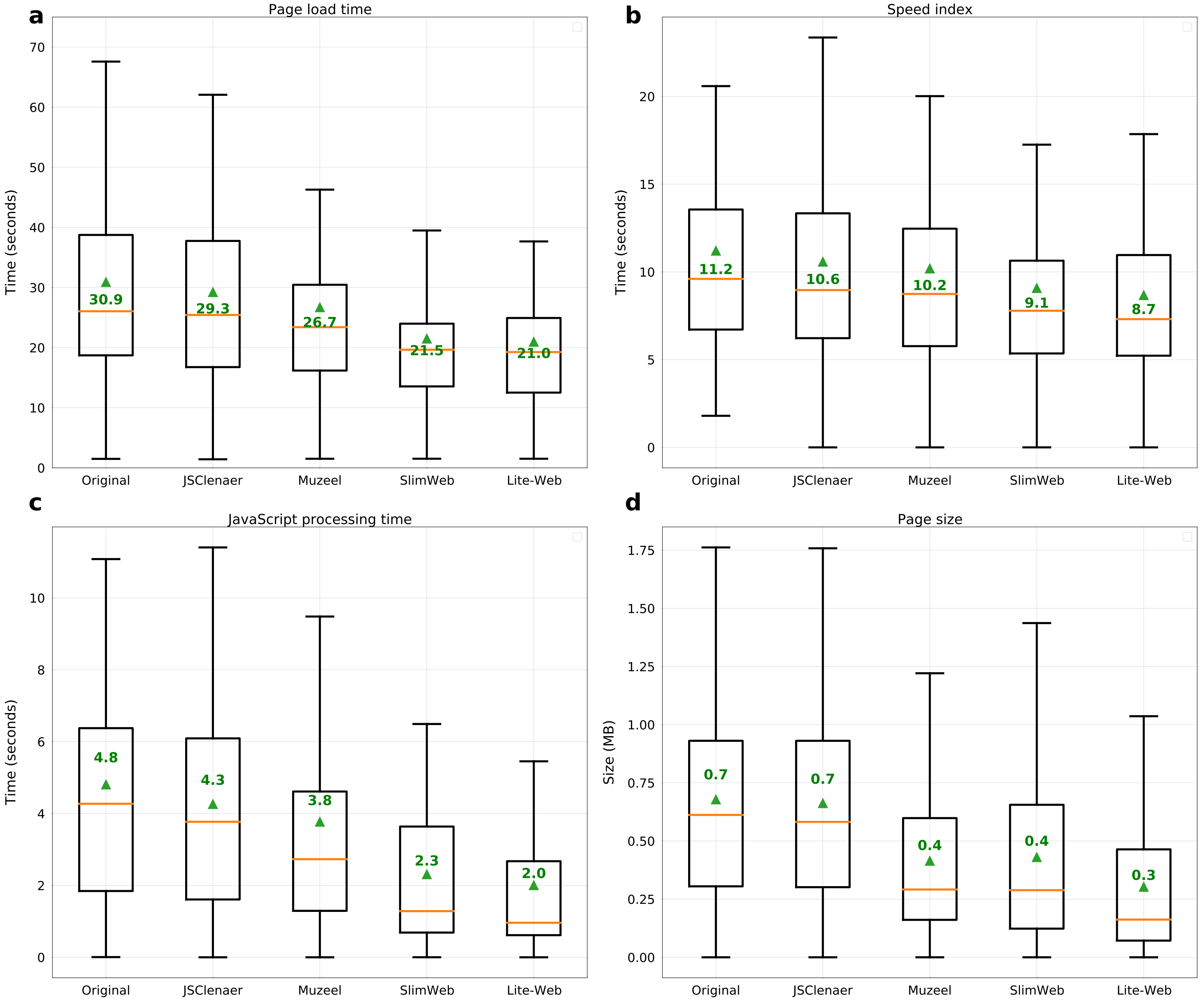

Fig. S5. The impact of Lite-Web and its constituent parts. Using the 100 Pakistani webpages most frequently visited in 2021, we compared the original version to the version produced by Lite-Web, as well as the versions produced by each of its constituent parts, namely SlimWeb, Muzeel, and JSCleaner. **a**, Evaluating page load time. **b**, Evaluating Speed Index. **c**, Evaluating JavaScript processing time. **d**, Evaluating page size.

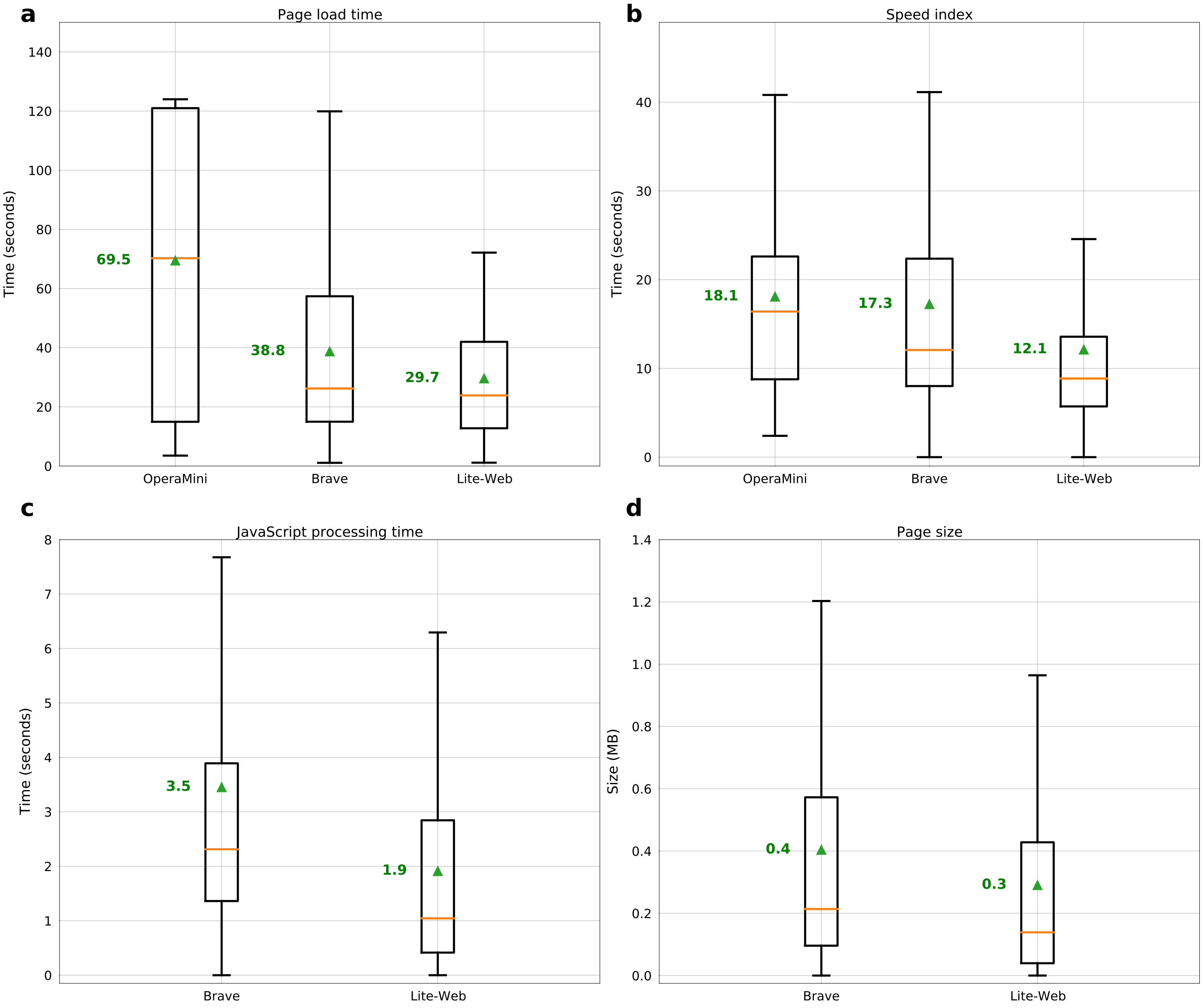

Fig. S6. Comparing Lite-Web to existing alternatives. Using the 100 most frequently visited Pakistani webpages in 2021 to compare Lite-Web to existing alternatives, namely Brave and OperaMini. The evaluation is done on the same low-end phone (QMobile i6i 2020) under the same cellular network conditions (SCOM 4G) in the city of Lahore, Pakistan. **a**, Evaluating page load time. **b**, Evaluating Speed Index. **c**, Evaluating JavaScript processing time. **d**, Evaluating page size.

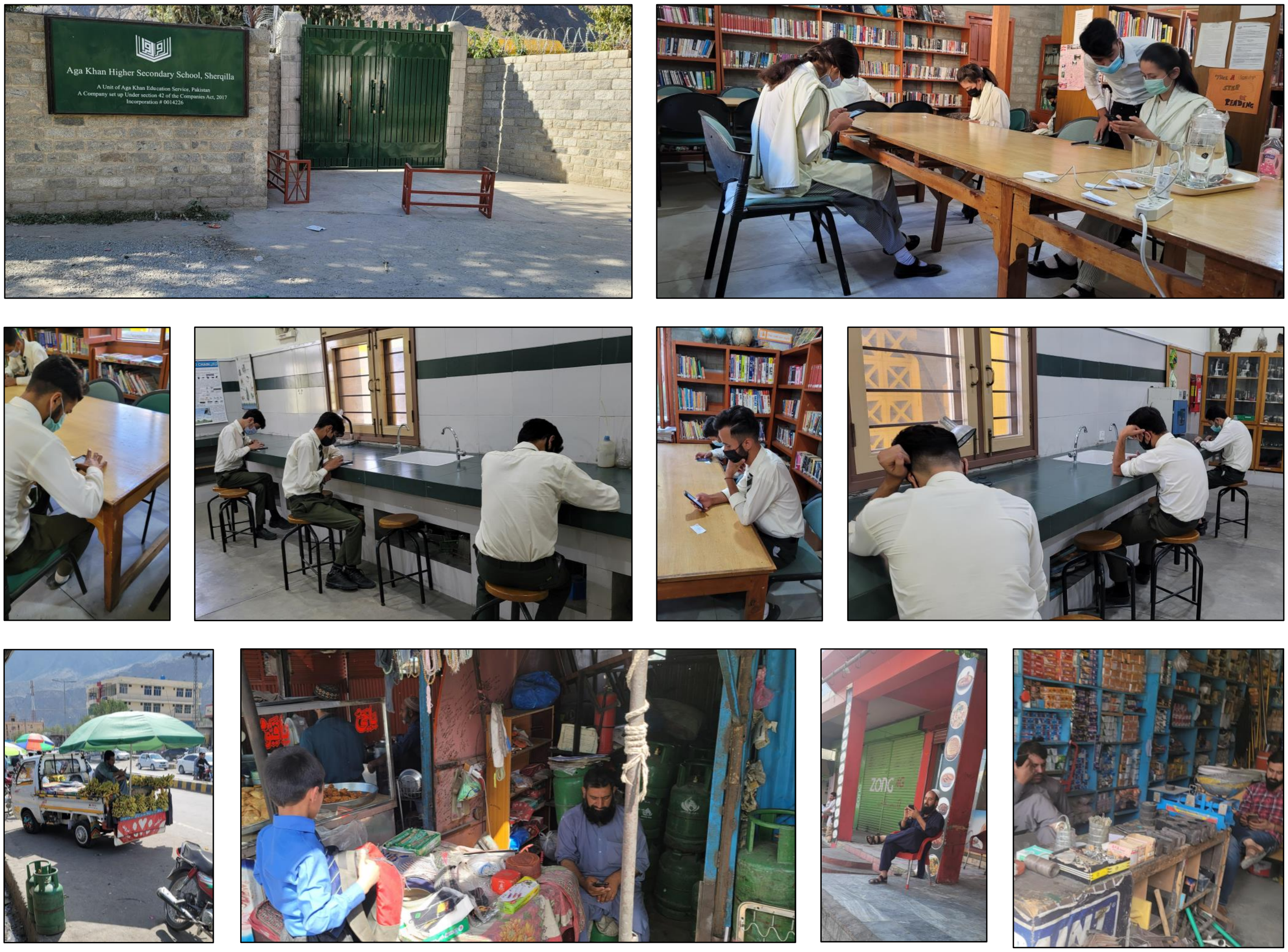

Fig. S7. The user study setup. All photos were taken during the user study that took place in the Gilgit-Baltistan province of Pakistan. The upper two rows show photos taken at one of the two schools where the experiment took place, showing how social distancing was observed while wearing masks. The bottom row shows photos of local merchants using their mobile phones.

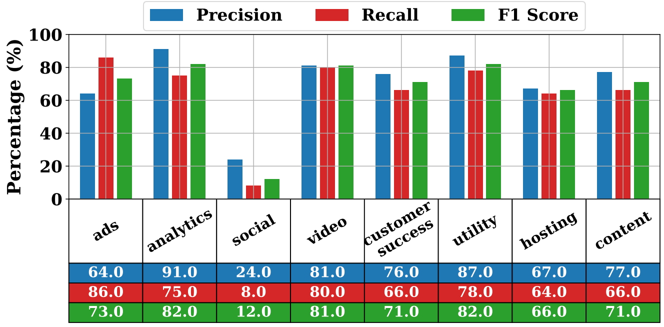

(a) K-Nearest Neighbors (KNN)

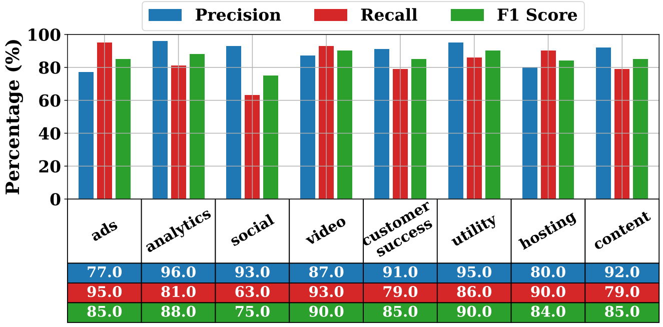

(b) Support Vector Machine

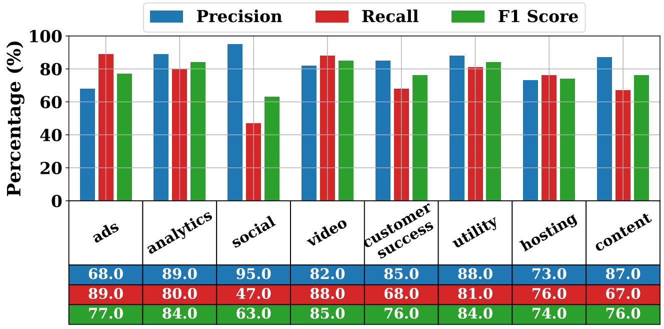

(c) Linear Support Vector Classifier (LSVC)

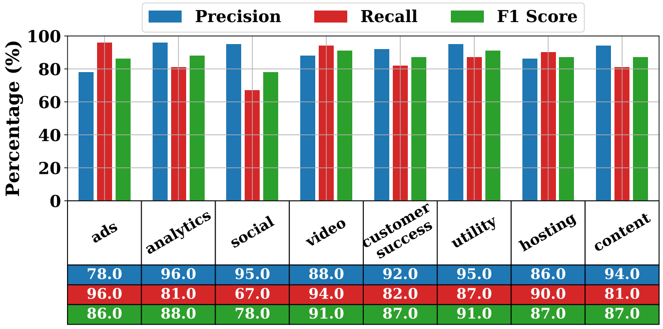

(d) Random Forest Classifier (RFC)

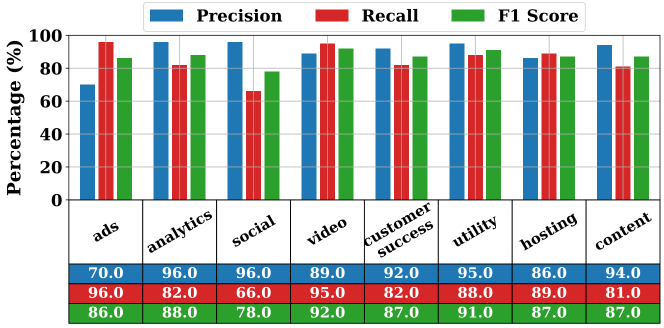

(e) XGBoost

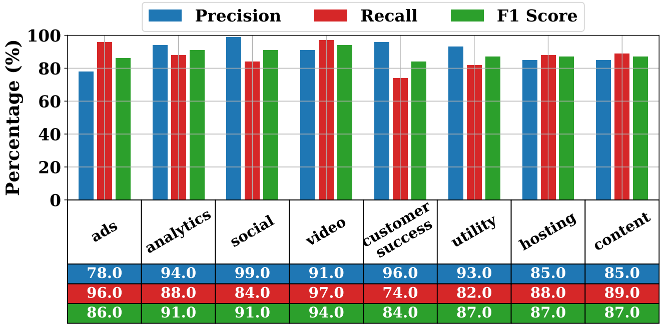

(f) Simple Neural Network

Fig. S8. For each of the 8 categories, the figure depicts the performance of different classifiers in terms of precision, recall, and F1-score.

Table S1. The cost per Gigabyte (measured based on the purchasing power parity) and the page load time (measured in seconds) in each of the locations considered in our study.

City/Country	Page Load Time	Cost
**Tokyo, Japan**	14.58	1.740530688
**Riga, Latvia**	13.69	2.166064982
**Hanoi, Vietnam**	11.77	1.365457547
**Sydney, Australia**	16.23	0.392413342
**Kuwait, Kuwait**	16.79	0.326203209
**Warsaw, Poland**	15.55	1.71009772
**Skopje, Macedonia**	14.49	4.737221997
**Mumbai, India**	20.61	1.429965586
**Ufa, Russia**	47.06	11.11507653
**Houston, United States**	16.21	0.910384068
**Fiji**	20.5	2.15010142
**Caracas, Venezuela**	26.17	32.06470695
**Beijing, China**	32.93	1.782011092
**Manama, Bahrain**	18.57	2.794117647
**Addis Ababa, Ethiopia**	34.9	9.616290019
**Seoul, South Korea**	19.43	1.214332492
**London, United Kingdom**	15.42	1.282051282
**Bremen, Germany**	14.79	2.388818297
**Ulaanbaatar, Mongolia**	16.54	1.49798178
**Sarvar, Hungary**	22.04	8.029204014
**Vancouver, Canada**	14.16	3.690753691
**Lahore, Pakistan**	20.89	3.963790563
**Gaggio Montano, Italy**	12.36	0.599739244
**Zenica, Bosnia and Herzegovina**	13.18	2.327365729
**Zilina, Slovakia**	23.37	1.95164076
**Cairo, Egypt**	19.2	2.303403756
**Islamabad, Pakistan**	18.28	3.963790563
**Bishkek, Kyrgyzstan**	15.94	0.328808135
**Dubai, UAE**	15.41	6.349206349
**Helsinki, Finland**	13.25	0.078794902
**Havana, Cuba**	18.76	43.33333333
**Accra, Ghana**	34.7	3.158195317
**Arkhangelsk, Russia**	17.24	11.11507653
**Kingston, Jamaica**	25.57	9.056284805
**Zaporizhzhya, Ukraine**	14.62	2.629174993
**Monterrey, Mexico**	21.4	5.576513538
**Copenhagen, Denmark**	12.64	0.400456216
**Tenerife, Spain**	15.71	0.910384068
**Moscow, Russia**	17.27	11.11507653
**Kumasi, Ghana**	20.4	3.158195317
**Arusha, Tanzania**	18.21	4.4428402
**Cebu City, Philippines**	25.88	4.4428402
**Namangan, Uzbekistan**	21.18	11.60614212
**Bogota, Colombia**	17.22	14.77002473
**Tegucigalpa, Honduras**	19.7	7.329089399
**Sarajevo, Bosnia and Herzegovina**	14.87	2.327365729
**Tainan, Taiwan**	16.52	2.878057597
**Bhaktapur, Nepal**	15.11	1.203421492
**Kiev, Ukraine**	17.36	2.629174993
**Incheon, South Korea**	14.14	1.214332492
**Rabat, Morocco**	15.03	2.234910277
**Kathmandu, Nepal**	20.86	1.203421492
**Nur-Sultan, Kazakhstan**	29.25	1.84994771
**Coimbatore, India**	30.26	1.429965586
**New York, United States**	16.61	0.910384068
**Riyadh, Saudi Arabia**	17.85	0.348027842

Table S2. Xiaomi Redmi Go specifications.

Category	Specifications
**Launch year**	2019
**CPU**	Quad-core 1.4 GHz Cortex-A53
**Operating System**	Android 8.1 Oreo (Go edition)
**Memory**	8GB 1GB RAM
**Price**	around 70 USD

Table S3. Specifications of the low-end phones used in the experiment that evaluates JavaScript over the years 2015-2020. Note that Xiaomi Redmi Go was released in January 2019, so we used it for 2018 since we were unable to obtain another low-end phone released in 2018.

Year	Low-end Phone	CPU	RAM
**2015**	HTC Desire 520	Quad-core 1.1 GHz Cortex-A7	1 GB
**2016**	Huawei Y3II	Quad-core 1.0 GHz Cortex−A53	1 GB
**2018**	Xiaomi Redmi go	Quad-core 1.4 GHz Cortex-A53	1 GB
**2019**	Alcatel 1C	Quad-core 1.3 GHz Cortex−A53	1 GB
**2020**	Nokia C1	Quad-core 1.3 GHz	1 GB

Table S4. Specifications of the high-end phones used in the experiment that evaluates JavaScript over the years 2015-2020.

Year	High-end Phone	CPU	RAM (GB)
**2015**	Samsung Galaxy S6 edge	Octa-core 4x2.1 GHz Cortex-A57, 4x1.5 GHz Cortex-A53	3
**2016**	Huawei P9	Octa-core 4x2.5 GHz Cortex-A72, 4x1.8 GHz Cortex-A53	4
**2017**	Samsung Galaxy S8+	Octa-core 4x2.3 GHz Mongoose M2, 4x1.7 GHz Cortex-A53	4
**2018**	Huawei Mate 20 Pro	Octa-core 2x2.6 GHz Cortex-A76, 2x1.92 GHz Cortex-A76, 4x1.8 GHz Cortex-A55	6
**2019**	Samsung Galaxy S10+	Octa-core 2x2.73 GHz Mongoose M4, 2x2.31 GHz Cortex-A75, 4x1.95 GHz Cortex-A55	12
**2020**	Samsung Galaxy S20	Octa-core 2x2.73 GHz Mongoose M5, 2x2.50 GHz Cortex-A76, 4x2.0 GHz Cortex-A55	8

Table S5. Supervised learning evaluation summary.

Models	Recall	Precision	F1-Score
KNN	0.75	0.76	0.75
SVM	0.86	0.88	0.86
LSVC	0.79	0.81	0.79
RFC	0.88	0.89	0.88
XGBoost	0.88	0.89	0.88
4-layer Neural Network	0.89	0.9	0.89

References

1. M Chaqfeh, et al., To block or not to block: Accelerating mobile web pages on-the-fly through javascript classification. *CoRR* **abs/2106.13764** (2021).
2. M Chaqfeh, Y Zaki, J Hu, L Subramanian, Jscleaner: De-cluttering mobile webpages through javascript cleanup in *Proceedings of The Web Conference 2020*. pp. 763–773 (2020).
3. Mozilla, individual contributors, Document object model (dom) (https://developer.mozilla.org/en-US/docs/Web/API/Document_Object_Model) (2005-2019).
4. Mozilla, individual contributors, The html dom api (https://developer.mozilla.org/en-US/docs/Web/API/HTML_DOM_API) (2005-2019).
5. E International, Ecmascript® 2018 language specification (http://www.ecma-international.org/ecma-262/9.0/index.html) (2019) Accessed: 2019-05-05.
6. H Archive, Third parties | 2019 | the web almanac by http archive (https://almanac.httparchive.org/en/2019/third-parties) (2019) Accessed: 2020-01-2.
7. P Hulce, third-party-web/entities.json5 at 8afa2d8cadddec8f0db39e7d715c07e85fb0f8ec · patrickhulce/third-party-web (https://github.com/patrickhulce/third-party-web/blob/8afa2d8cadddec8f0db39e7d715c07e85fb0f8ec/data/entities.json5) (2019) Accessed: 2020-01-2.
8. T Chen, C Guestrin, Xgboost: A scalable tree boosting system in *Proceedings of the 22nd acm sigkdd international conference on knowledge discovery and data mining*. pp. 785–794 (2016).
9. AF Agarap, Deep learning using rectified linear units (relu). *arXiv preprint arXiv:1803.08375* (2018).
10. J Kupoluyi, et al., Muzeel: Assessing the impact of javascript dead code elimination on mobile web performance in *Proceedings of the 22nd ACM Internet Measurement Conference*, IMC '22. (Association for Computing Machinery, New York, NY, USA), p. 335–348 (2022).
11. NG Obbink, I Malavolta, GL Scoccia, P Lago, An extensible approach for taming the challenges of javascript dead code elimination in *2018 IEEE 25th International Conference on Software Analysis, Evolution and Reengineering (SANER)*. (IEEE), pp. 291–401 (2018).
